# Are interventions effective at improving driving in older drivers?: A systematic review

**DOI:** 10.1186/s12877-020-01512-z

**Published:** 2020-04-03

**Authors:** H. I. Castellucci, G. Bravo, P. M. Arezes, M. Lavallière

**Affiliations:** 1 Centro de Estudio del Trabajo y Factores Humanos, Escuela de Kinesiología, Facultad de Medicina, Universidad de Valparaíso, Valparaiso, Chile; 2 Facultad de Ciencias de la Salud, Universidad de Las Américas, Providencia, Chile; 3 ALGORITMI Centre, School of Engineering of the University of Minho, Guimarães, Portugal; 4 Module de Kinésiologie, Département des Sciences de la Santé, Université du Québec à Chicoutimi (UQAC), Saguenay, QC Canada; 5 Laboratoire de recherche biomécanique & neurophysiologique en réadaptation neuro-musculo-squelettique - Lab BioNR, UQAC, Saguenay, QC Canada; 6 Centre intersectoriel en santé durable – UQAC, Saguenay, QC Canada; 7 Centre de recherche-Charles-Le Moyne-Saguenay–Lac-Saint-Jean sur les innovations en santé (CRCSIS), Longueuil, Canada

**Keywords:** Elderly drivers, Road safety, Prevention, Collisions

## Abstract

**Background:**

With the aging of the population, the number of older drivers is on the rise. This poses significant challenges for public health initiatives, as older drivers have a relatively higher risk for collisions. While many studies focus on developing screening tools to identify medically at-risk drivers, little research has been done to develop training programs or interventions to promote, maintain or enhance driving-related abilities among healthy individuals. The purpose of this systematic review is to synopsize the current literature on interventions that are tailored to improve driving in older healthy individuals by working on components of safe driving such as: self-awareness, knowledge, behaviour, skills and/or reducing crash/collision rates in healthy older drivers.

**Methods:**

Relevant databases such as Scopus and PubMed databases were selected and searched for primary articles published in between January 2007 and December 2017. Articles were identified using MeSH search terms: (“safety” OR “education” OR “training” OR “driving” OR “simulator” OR “program” OR “countermeasures”) AND (“older drivers” OR “senior drivers” OR “aged drivers” OR “elderly drivers”). All retrieved abstracts were reviewed, and full texts printed if deemed relevant.

**Results:**

Twenty-five (25) articles were classified according to: 1) Classroom settings; 2) Computer-based training for cognitive or visual processing; 3) Physical training; 4) In-simulator training; 5) On-road training; and 6) Mixed interventions. Results show that different types of approaches have been successful in improving specific driving skills and/or behaviours. However, there are clear discrepancies on how driving performance/behaviours are evaluated between studies, both in terms of methods or dependent variables, it is therefore difficult to make direct comparisons between these studies.

**Conclusions:**

This review identified strong study projects, effective at improving older drivers’ performance and thus allowed to highlight potential interventions that can be used to maintain or improve older drivers’ safety behind the wheel. There is a need to further test these interventions by combining them and determining their effectiveness at improving driving performance.

## Background

The number of older drivers is rapidly rising due to the aging population [1–4]. It is projected that, by 2030, 20% of the population will be 65 years or older [5]. In Canada, it is expected that, by 2026, 1 driver out of 5 will be 65 years or older [6]. With increasingly active lifestyles, seniors are expected to rely even more on their vehicles, taking more trips, driving further distances, and keeping their licenses longer than prior generations [7]. In fact, it is anticipated that a large proportion of both men and women will continue driving well into their 80’s [8]. For example, a majority of Canadian seniors hold a valid driver’s license (4.7 million in 2017. representing 75% of all seniors) [9]. These trends pose significant public health concerns, as older drivers are disproportionately involved in collisions [9] causing serious injury and death, when exposure (kilometres driven) is taken into account [10].

The higher crash rates in older adults may be due to age-related medical conditions. For example, seniors may develop vision impairment [11, 12], mild cognitive impairment, early dementia, Parkinson’s disease and other neurodegenerative disorders, or may have suffered a stroke. All these conditions produce symptoms that impair the skills that are required to drive safely. Many studies show that these conditions lead to worse driving performances both on-road [13, 14] and in-simulator evaluations [15] compared to the general older adult population. Despite these numerous health conditions associated with aging that might negatively impact driving performance, the proportion of older drivers who are considered healthy still represents the largest segment of these drivers. Therefore, there is a need to assess interventions that are tailored for them.

Multiple assessment tools are available for use within clinical settings to screen for at-risk drivers. Although many assessments/tools are quick and easy to administer, a screening battery has not yet been developed [16, 17]. The potential to detect unsafe drivers versus successfully identifying safe drivers is an important consideration, particularly as removing one’s license can have negative consequences [18, 19]. Prior studies have found that driving cessation is associated with increased depression, social isolation, institutionalization and even early mortality [20, 21]. A recent survey conducted by Vrkljan et al. [22] showed inconsistency in practice among evaluations in a sample of driver assessment centres for medical fitness to drive (*n* = 47). Their results highlight the necessity of evidence-based guidelines for the training and assessment of at-risk drivers.

While licensing authorities must consider public safety when delivering driver’s licenses, it is important to help seniors drive for as long as possible to facilitate their autonomy and independence. This is particularly the case since there are few programs to help seniors adjust to non-driving.

Alternatively, interventions aimed at improving or maintaining driving skills offer new opportunities to help seniors drive safer, longer. Several studies have examined the impact of workbooks, seminars, and cognitive, simulator or on-road training on driving performance in older adults in general, and in those with various medical conditions. The purpose of this systematic review is to synopsize the current literature on interventions that are tailored to improve driving: Self-awareness, knowledge, behaviour, skills and/or reducing of the number of collisions in healthy older drivers.

## Methods

A systematic literature review (SLR) methodology [23] was used to synopsize the current literature on interventions that are tailored to improve older individuals’ driving. This methodology is scientifically transparent, replicable, and useful to generate an in-depth analysis of the scientific literature [24]. An initial exploratory review was produced prior to conduct the full SLR [25]. This method allows to elucidate common knowledge of the topic, to identify if the proposed SLR fits the existing knowledge in the area, to determine the key concepts and to refine the research question. Also, this SLR followed a five-step approach proposed by Denyer and Tranfield [25]: 1) Question formulation; 2) Locating studies; 3) Study selection and evaluation; 4) Analysis and synthesis; and 5) Reporting and using the results. Based on witch, a review protocol was used regarding the formulation of the research question, on the selection of scientific databases and search terms, and on the inclusion and exclusion criteria for searching and analysing retrieved publications.

### Step 1: question formulation

A PICO framework (Population, Intervention, Control, Outcomes) was used to generate the research question of this study (Step 1). This approach allows for a more systematic approach regarding the identification of relevant information and its understanding by using these four categories [24, 26]. Therefore, the research question formulated for this SLR was: In healthy older drivers (P), which type of intervention program (I), education, computer-based, physical training, on-road, simulator-based or mixed program (C) improved driving: Self-awareness, knowledge, behaviour, skills and/or crash rates (O)?

### Step 2: locating studies

Based on the research question defined in Step 1, search strings to be used and appropriate bibliographic databases were defined in Step 2. *Scopus* and *PubMed* databases were used as they encompass a wide array of scientific areas as well as the most relevant peer-reviewed publications [27]. Articles were identified using MeSH search terms and strings (in English only): (“safety” OR “education” OR “training” OR “driving” OR “simulator” OR “program” OR “countermeasures”) AND (“older drivers” OR “senior drivers” OR “aged drivers” OR “elderly drivers”). The EndNote version X9.2 management software package was used to manage all the information.

### Step 3: study selection and evaluation

To select the most relevant scientific articles to include in the review, the inclusion and exclusion criteria were defined in Step 3. The following key inclusion criteria were defined prior to the search:

- Original articles written in English and published in peer-reviewed journals;
- Published or in press between January 2007 and December 2017.
- Articles were excluded if the sample presented drivers with specific health conditions (e.g. traumatic brain injury, vision impairment associated with specific pathologies, stroke or Parkinson’s Disease).

Titles and abstracts of papers were scanned independently by three of the authors to identify relevant articles to retrieve for full text analysis. In cases where the papers seemed potentially eligible, but no abstract was available, the full text of the paper was retrieved. Disagreements between authors led to a deeper joint analysis of the paper; and a decision was then made regarding its inclusion. Full texts were independently reviewed for inclusion by the same three authors.

The literature search purposely only included studies between 2007 and 2017, since previous systematic reviews on the topic had already been conducted [28, 29]. These reviews cover researches completed prior to 2008, and despite being well-conducted systematic reviews, more recent studies on different interventions to improve older drivers’ performance have been conducted but not yet been synthesized. To our knowledge, there is no more recent review in the literature, despite the need for guiding evidence-based practices.

### Step 4: analysis and synthesis

Step 4 consisted in analysing, extracting and managing papers’ information to identify and highlight key components of the research conducted and its results. Primary studies meeting the inclusion criteria and reported in the included reviews were identified, and the corresponding data was extracted using a standardized data extraction form. The Quality Assessment Tool set known as “QualSyst tools” was selected as it allows appraisal of quality while assessing potential bias over a wide variety of research designs, from experimental to observational [30]. Furthermore, this set of tools has one version for quantitative studies and another one for qualitative studies, and in this review, the first one was used. The quantitative version consists in a checklist of 14 questions, with possible answers of: yes, no, partial or not applicable. The score for a “yes” answer is 2 points, for a “partial” answer 1 point, and for “no” 0 points. The sum of all answers is then calculated from the corresponding points and divided by the total of applicable responses.

The QualSyst was used by three of the authors to evaluate internal and external validity of the considered studies. The QualSyst tool was originally created as a threshold allowing a study to be included in a review through a cut-off point (0.55 to 0.75) [30]. In this review, the QualSyst cut-off score of 0.55 was chosen to capture 75% of the articles initially deemed as relevant for the review, as well as to ensure the inclusion of several descriptive articles containing valuable data [31]. More specifically, papers with a score higher than 0.8 were classified as having a strong methodology (> 0.8), between 0.79 and 0.71 as being good, and adequate if the score was between 0.7–0.55, or limited and therefore excluded if the score was lower than 0.55 [32, 33].

By using an approach adapted from Sackett et al. [24], identified papers were also categorized using a standardized value system to grade biomedical practices according to the following system:

- Level I: Systematic reviews, meta-analyses, randomized controlled trials
- Level II: Two groups, nonrandomized studies (e.g., cohort, case control)
- Level III: One group, nonrandomized (e.g., before and after, pre-test and post-test)
- Level IV: Descriptive studies including analysis of outcomes (e.g., single-subject design, case series)
- Level V: Case reports and expert opinions including narrative literature reviews and consensus statements.

Using such an approach while conducting a review also provides a scheme of references for the clinicians interested in using such methods/approaches in their practicum. Evidence-based practices are built on the assumption that scientific evidence of the effectiveness of an intervention can be deemed more or less strong and valid according to a hierarchy of research designs, the assessment of the quality of the research, or both.

### Step 5: reporting and using the results

For Step 5, the results were grouped (Tables 1, 2, 3, 4, 5, 6, 7 and 8) according to the specific type of programs (independent variables) used by Golisz [59], who considered 5 different options such as: (1) education-based training programs, (2) computer-based training, (3) physical training, (4) simulator-based training, and (5) route-based or actual driving training. Moreover, another independent variable was considered in the current study (6 - Mixed programs) since there were many investigations that used two types of interventions, therefore making it difficult to differentiate which one of the variables is responsible for the obtained results. It is noteworthy that the route-based or actual driving training (5) alone was not used in any of the studies evaluated and is therefore not presented in the tables.

**Table 1:** Summary of the reviewed studies

Type of Programs	Method used to collect the dependent variables						
	On-road (OR)	Simulator (S)	Test/Questionnaire (TQ)	OR/TQ	S/TQ	OR/S	Total
Education	1	0	4	1	0	0	6
Computer based	1	1	4	0	1	0	7
Physical training	2	0	0	0	0	0	2
Simulator-based training	0	2	0	0	0	0	2
Mixed	1	0	0	4	0	3	8
**Total**	**5**	**3**	**8**	**5**	**1**	**3**	**25**

**Table 2:** Summary of the dependent variables considered in the reviewed studies

Dependent variables					
Type of Programs	Self-Awareness and/or Knowledge (SA/K)	Behavior (B)	Skills (S)	Crash Rates (CR)	Total
Education	2	4	0	1	**7**
Computer based	0	2	4	1	**7**
Physical training	0	0	2	0	**2**
Simulator-based training	0	0	2	0	**2**
Mixed	2	4	4	0	**10**
**Total**	**4**	**10**	**12**	**2**	**28**

Each study may have had more than one type of dependent variable, explaining why the totals add up to more than 25

**Table 3:** Summary of the results

	Dependent variables														
Type of Programs	Self-Awareness and/or Knowledge (SA/K)			Behavior (B)			Skills (S)			Crash Rates (CR)			Total		
	(+)	(−)	(+/−)	(+)	(−)	(+/−)	(+)	(−)	(+/−)	(+)	(−)	(+/−)	(+)	(−)	(+/−)
Education	2	0	0	1	2	1	0	0	0	0	1	0	3	3	1
Computer based	0	0	0	1	1	0	3	1	0	0	0	1	4	2	1
Physical training	0	0	0	0	0	0	2	0	0	0	0	0	2	0	0
Simulator-based training	0	0	0	0	0	0	1	1	0	0	0	0	1	1	0
Mixed	2	0	0	4	0	0	3	1	0	0	0	0	9	1	0
**Total**	**4**	**0**	**0**	**6**	**3**	**1**	**9**	**3**	**0**	**0**	**1**	**1**	**19**	**7**	**2**

Each study may have had more than one type of dependent variable, explaining why the totals add up to more than 25

**Table 4:** Synthesis of intervention studies involving education-based training programs

Authors	Number (n), Age (yr) Country (c)	R.D.	Objective	Study description	Dependent Variable	Relevant results	QS
Coxon et al., [34]	*n* = 366 yr = between 75 and 94 years old. c = Australia	RCT	To ascertain whether a safe-transportation program can change driving exposure while maintaining community participation of older drivers	Participants were randomized in 2 groups: -Intervention group (n: 190): They had to participate in two sessions held 1 month apart. The session was delivered by an occupational therapist face to face and lasted 120 and 45 min, the first and second session, respectively -Control group (n: 190): Did not receive any education. Before the randomization all the participants performed the baseline assessment and self-reported questionnaires. Also, the driving exposure was measure over the 12 months of study through an in vehicle monitoring device.	(B) Driving exposure measure over the 12-month study period with an in-vehicle monitoring device (B): Driving space and use of alternate transportation were measured using a questionnaire. (B): Depressive symptoms were measured using the Geriatric Depression Scale.	B (−): An education program does not translate to significant differences in the distance driven per week, the restriction of driving space, the use of alternate transportation and the community participation after 12-month post intervention. Also, there was no difference between the control and intervention group in proportion of participants with two or more depressive symptoms at 12 months. B (+): Participants in the intervention group were more likely to be closer to adopting self-regulatory driving practices at 12 months than control group. The odds of the participants in the intervention group being in a higher behavioral profile were 1.6 times greater than those in the control group.	0,89
Nasvadi and Vavrik, [35]	*n* = 139 (was considered phase 2) yr = between 55 and 94 years old. c = Canada	Retro- Cohort	Determine if the crash rate of aging drivers can be mitigated by post-license driver education	The participants were divided in 2 group: -Drivers who attended the 55 alive/mature driving courses between January 1, 2000 and July 31, 2003 (n: ns). -Drivers who did not attend the educational program (n: ns). Then were compared the crashes rates after the date of attendance at the course, until December 31, 2003	(CR): Crashes and violations were obtained from Insurance Corporation of British Columbia.	CR (−): Older men and women who attended the 55 Alive/Mature Driving course had a 1.5 times greater odds of being involved in a crash than their matched controls. These results were marginally significant (*p* = .078). For women separately, there was no difference between subjects and controls for the number of post-course crashes, regardless of age category. However, for men, drivers aged 75 years and older who attended 55 Alive/Mature Driving were 3.8 times more likely to be involved in a crash (*p* = .050).	0,81
Nasvadi, [36]	*n* = 367 yr = between 55 and 94 years old. c = Canada	Retro- Cohort	Examine long-term learning outcomes of a sample of older drivers who attended a mature driver education program.	The cohort consisted of drivers aged 55 years and older who attended the 55 alive/mature driving course. All participants were surveyed by telephone.	(SA/K) and (B): The survey contained open-ended and closed questions and asked respondents to recall what they had learned in the course, and how their driving behavior had changed because of attending.	SA/K (+) B (+): Three quarters of participants said they changed their driving habits as a result of attending the course (55 alive/mature) including: increased awareness and visual skills; changes in attitude; improved speed and space margins; avoidance of hazards; using more caution; obeying road rules; and improved vehicle maneuvers. Men were more likely to report their driving skills had improved since taking the course, and older men reported significantly higher mean comfort scores with their driving.	0,79
Jones et al., [37]	*n* = 58 yr = average age 70.9 years old (ages N/S). c = USA	RCT	Describe driving experiences and habits of a community sample of older drivers (60+ years) and to determine whether the program reduces these older adults’ driving risk exposures.	Participants were randomized in 2 groups: -Intervention group (n: 33): 4 weeks of training in a classroom setting with 2 h of training per week. The 4 sessions include: roadwise review, road smart, safer driving and being medwise to stay roadwise medication. -Control group (n:25): Did not receive training The both group completed the baseline questionnaire and after the 4 weeks completed the questionnaire again.	(B): Driving habit risk exposure were divide in two types -Higher driving risk exposure: defined as frequency of driving further than 10 miles from home, after dark, between 5 pm and 7 pm, and on interstates. -Lower driving risk exposure: defined as frequency of driving less than 2 miles from home and before 9 am. (B): They also asked for driving experience	B (−): There were no statistical differences in lower and higher driving risk exposure when comparing the intervention and control group.	0,79
Gaines et al., [38]	*n* = 195 yr = between 79 and 84 years old c = NM	RCT	Assess the process and short-term effects of the CarFit program.	Participants were randomized in 2 groups: -CarFit group (n: 83): the intervention was carried out in one day through an individual appointment with the community’s CarFit event coordinator. Each CarFit assessment required approximately 15 min for completion. Therefore, the total time could be 150 min. -Comparison group (n:112): Did not receive training All the participants answered the driving questionnaire in the baseline and six months after the CarFit intervention.	(B): A driving questionnaire based in 3 parts was applied: -Driving Activity: a higher score indicates greater driving activity. -Comfortable Driving: a lower score indicates greater comfort during the driving activities. -Driving Behaviors: a lower score indicates safer driving behaviors.	B (−): There was no statistically significant difference between having a CarFit intervention or not receiving training after the six-month post CarFit intervention in driving behaviors.	0,75
Jones et al., [39]	*n* = 44 yr = average age 79 years old (ages N/S). c = USA	RCT	Compare the impact of a multi-session interactive, expert-led version of the training program (Seniors on the MOVE – Version-A) to a self-guided and less resource intensive version of the program (Seniors on the Move – Version-B) on older drivers’ knowledge and behavior pertaining to driving.	Participants were randomized in 2 groups: -SOM-A (n: 20): Consisted in the four sessions utilized in the Jones et al., 2011 and the CarFit assessment. -SOM-B (n: 24): Consisted in a self-guided and less intensive version of the SOM-A. The participants had to assist to a one session and two optional (one was in class and the other the CarFit assessment) At the beginning of each session the groups completed the baseline questionnaire, then immediately after the completion of the programs and finally after 6 months after the baseline.	(SA/K): Self-reported driving knowledge 15 items were developed by the authors to assess specific details taught during the sessions.	SA/K (+): They found significant differences in SOM-A group between the baseline and the first follow up in the knowledge of the proper placement of the head restraint, the time checking tire pressure, muscle relaxers do not affect driving and the definition of moderate drinking for older adults. But this significant difference with the follow up two was only with the item of definition of drinking for older adults. For the other hand, the SOM- B demonstrated a significant difference between follow up two on knowledge about muscle relaxers. SA/K (+/−): Comparing the mean total knowledge scores baseline and immediately after the completion of the training between the groups they found that the program with 4 obligatory sessions was significant greater than the self-guided program with only one required session. However, this difference was not significant after 6 months.	0,68

**Table 5:** Synthesis of intervention studies involving computer-based training

Authors	Number (n), Age (yr) Country (c)	R.D.	Objective	Study description	Dependent Variable	Relevant results	QS
Edwards et al., [40]	*n* = 500 yr = average age 74 and 75 years old (ages N/S). c = USA and UK	Cohort	The current analyses were conducted to examine whether completing this speed of processing training regimen delays driving cessation.	The cohort was formed by participants from 2 different study. The intervention was based in 10 speed of processing training sessions led by a trainer in which the subjects practiced computerized exercises of visual attention aimed at enhancing the speed and accuracy of visual performance. The sessions lasted 1 h, twice a week for 5 weeks. The assessment carried out at baseline, immediately post training, and was repeated 3 years after training.	(B): Driving status and the number of days per week driven was evaluated with the Mobility Driving Habits Questionnaire. -Far visual acuity was evaluated with a standard letter chart. -Mental status was assessed with the Mini-Mental State Examination.	B (+): Speed of processing training participation was protective against driving cessation, mainly in those drivers who drove more often and those with better vision. Thus, the participants who completed the training were 40% less likely to cease driving across the subsequent 3 years as compared with controls	0,86
Ball et al., [41]	*n* = 908 yr = between 65 and 91 years old. c = USA	RCT	To test the effects of cognitive training on subsequent motor vehicle collision (MVC) involvement of older drivers.	Participants were randomized in 4 groups: -Control group (n:298): No training -Memory training (n: 103): Based in mnemonic strategies. -Reasoning training (n: 133): Based in strategies for finding the pattern in a letter or word series and identifying the next item in the series. -Speed of processing training (n: 129): based in practice of visual attention skills and the ability to identify and locate visual information quickly in increasingly demanding visual displays. The sessions were led by trainers, conducted in groups of 2–4 participants during approx. 70 min sessions over a period of 5 to 6 weeks. In each intervention condition, 10 training sessions were carried out twice a week over a 5-week period Participants completed assessments at baseline, immediately following, and annually at 1, 2, 3, and 5 years.	(CR): The primary outcome was state-recorded motor vehicle collision (MVC) obtained from the Departments of Motor Vehicles in the states of Alabama, Indiana, Maryland, and Pennsylvania. The variable were: Total collisions, at-fault collisions, person-time (in years), person-miles, at-fault crashes/year, at-fault crashes/mile rate ratios. Mileage—The number of miles driven per week was reported by participants on the Mobility Driving Habits Questionnaire and was used to calculate the dependent variable of interest, rate of MVCs per person mile driven	CR (−): The participants who carried out the memory training do not show a significant association in the reduction of rate of at fault MVC per year CR (+): The participants under speed processing training and reasoning training experienced a significantly lower rate of at fault MVC per year of driving exposure or per person mile driven.	0,86
Horswill et al., [42]	*n* = 75 yr = between65 and 89 years old. c = Australia	RCT	Examine the longer-term effects of hazard perception.	Participants were randomized in 3 groups: -Training without booster (n: 26): performed the hazard perception training that consisted in an instructional video followed by video-based exercises. -Training with booster (n: 25): After one month of receive the same training of the group without booster they received 22 min of additional training video. -Placebo (n: 24): they had a placebo intervention watching another video with clips of a driving instructor discussing aspects of safe driving. All the groups performed the hazard perception test in the first session prior to the training and then after one and three-month post intervention.	(S): Simple spatial RT test: the participants must touch as quickly as possible 15 high contrast rectangles that appeared one after another on the computer screen at random intervals.(S): Hazard perception tests: 4 hazard perception tests per participant were generated from a pool of 153 videos filmed from the driver’s perspective.	S (+): The participants that carried out the training responded 0.81 s faster than baseline compared with those in the placebo condition. This difference it was maintained after one and three months of following with 0,67 s and 0,45 s faster, respectively. S (+): The participants who were under intervention had a significant immediate effect of training on hazard perception S (+/−): The hazard perception training with booster did not show a significant difference relative to baseline than training without booster based on the hazard perception test scores.	0,79
Edwards et al., [43]	n = 500 yr = average age 72.08, 74.13 and 74.52 years old (ages N/S). c = UK	RCT	To examine how cognitive speed of processing training affects driving mobility across a 3-year period among older drivers.	Based upon their UFOV test performance the participants were randomized in 2 groups: -Cognitive speed of processing training (n: 66): the tasks in the computer involved identified and localize visual and auditory targets. -Computer contact internet training (n: 68): participants received instructions on computer hardware, how to use a mouse, how to use and e-mail and how to access and use web pages. The intervention had 10 sessions, 60 min in duration, guided by a trainer and involving 1–3 participants per class. Once finalized the training follow up interviews occurred within 3 years +/− 3 months of the participants last assessment.	(B): Driving behaviors was assessed with the Mobility Questionnaire: -Driving exposure: Total number of challenging conditions encountered while driving-Driving space: Extent into environment driven-Driving difficulty 3 (Alone, Lane and changes): Left-hand: Rating of difficulty while driving in each situation; 1 = no difficulty to 4 = extreme difficulty.-Driving difficulty 5 (Rush hour, High traffic, Night, Rain and Merging into traffic): Rating of difficulty while driving in each situation; 1 = no difficulty to 4 = extreme difficulty.	B (+): The participants that did not receive the speed of processing training experienced steeper decline in driving mobility across the 3-year period relative to the reference group as indicated by increased driving difficulty and decreased driving exposure and space. B (−): The participants that completed the speed of processing training experienced increased driving difficulty across time when driving alone, making lane changes, and making left-hand turns across oncoming traffic than did the reference group (driving difficulty three-item composite). B (−): The participants that were trained did not differ across time from the reference group in driving exposure, driving space, or the degree of driving difficulty as indicated by the five-item composite	0,79
Cuenen et al., [44]	*n* = 56 yr = average age 70.84, 69.84 and 73.06 years old (ages N/S). c = Belgium and Holland	RCT	The purpose of the present study was to investigate the effect of a computerized WM training on aspects of older drivers’ cognitive ability and driving ability.	Participants were randomized in 2 groups and the control group was collected: -Adaptative Training Group (n: 19): the difficulty level of the training was automatically adjusted on a trial-by-trial basis. -Non Adaptative Training Group (n:19): the difficulty level of the training was not adjusted -Control Group (n:18): No Training The two-training intervention group consisted in working memory training based in 3 subtasks: visuo-spatial task, a backward digit span task and a letter span task. The training was conducted at home, on a PC, via the internet with a total number of sessions between 20 and 25. After the training the participants developed the post-test that was the same pre-test and consisted in cognitive tasks and driving in a simulator scenario.	(S): Three Cognitive measures were evaluated: -Working memory -Attention -Inhibition (S): Six specific driving measures were evaluated: -Driving speed (km/h) -SDLP (m) -Gap acceptance (s) -Complete stops at stop signs -Giving right of way -Crashes (number)	S (+): The participants under computer training achieved a significant difference for working memory and the driving measure of giving right of way. In particular, participants who not were under training had lower working memory capacity and gave less right of way than the other two training groups. However, there was an improvement in the adaptive training group in cognitive ability, smaller in the non-adaptive training group and only minimal in the no-training control group supported for working memory. S (−): The effects of the training did not achieve a statistically significant difference for the cognitive abilities of attention and inhibition. S (+): The driving abilities such as driving speed and complete stops at stop signs achieved only marginally a significant effect. However, the other driving measures such as SDLP, gap acceptance, giving right of way, and crashes did not find statistically significant difference.	0,71
Cassavaugh and Kramer, [45]	*n* = 21 yr = average age 71.7 years old (ages N/S). c = USA	Pre-Post test	The present study’s main objective was to investigate whether training in laboratory tasks would transfer to driving performance in older adults.	All the participants were under the same computer-based training. The intervention consisted in 8-training session lasted 90 min and carried out in different days. The program had different tasks (attention, visuo-spatial working memory, manual control and dual tasks). The assessment consisted in two initial driving in simulator and two final post-intervention driving simulator session, identical to the first.	(S): Response accuracy and response time were measured in the selective attention and N-back tasks. Root mean square tracking error and time-on-target were analyzed for the tracking task. -Tracking task -Visual selective attention task -Visual–spatial N-back task -Dual tasks (S): Driving Simulator -Lane position and following distance were assessed in terms of root mean square error.-Response time to lead-vehicle brake events was measured in milliseconds.	S (+): The participants who were under a computer-based training achieved improvements in single and dual cognitive tasks. These improvements were translate to an improvement in driving simulator performance across the course of the study	0,69
Johnston et al., [46]	*n* = 53 yr = average age 68.83 years old (ages N/S). c = Canada	NRCT	The current study assessed the effectiveness of DriveSharp in training older drivers in a naturalistic class setting	The participants were divided in 2 groups: -Control group (n:18) -Experimental group (n:24) The intervention was the Drivesharp course that lasted 5 weeks with 2 sessions for week and each session was led by a facilitator with a duration of 60 min. This course was developed in a classroom environment on individual desktop computer with 3 games that incorporates divided attention and multiple object, intended to enlarge the UFOV and trains speed of processing. All participants completed trails that assess visual search, memory, and attention and a short version of the Hazard Perception Test in the pre-testing. After the 5 five weeks of training the participants attended the post-testing session that was the same pre-testing with only one difference that the experimental group completed a usability questionnaire.	(S): A brief version of the Hazard Perception Test was utilized (S): Trails A and B: were utilized to measure the processing speed, working memory, and executive control.	S (−): After the five weeks of training the analysis of performance data did not revealed any significant benefits to the Drivesharp course.	0,68

**Table 6:** Synthesis of intervention studies involving physical training

Authors	Number (n), Age (yr) Country (c)	R.D.	Objective	Study description	Dependent Variable	Relevant results	QS
Marottoli et al., [47]	*n* = 178 yr = average age 77.4 and 77.2 years old (ages N/S). c = USA	RCT	To determine whether a multicomponent physical conditioning program targeted to axial and extremity flexibility, coordination, and speed of movement could improve driving performance among older drivers.	The participants were randomized in 2 groups -Control group (n: 90): they received monthly in-home education modules reviewing general safety issues about home safety, fall prevention, and vehicle care. -Intervention group (n: 88):12 weeks of daily training of 15 min at home participants. The participants received a manual with images and instructions. Also, they had a weekly visit by a physical therapist to review the exercises. All the participants performed the baseline assessment and then the change in on-road driving performance (primary outcome) at 3 months was measured.	(S): Change in on-road driving performance at 3 months relative to baseline. (S): Secondary outcomes were the driving evaluators overall rating and number of critical errors at 3 months	S (+): The participants after the 12 weeks of daily training at home in the intervention group maintained the driving performance meanwhile in the control group they declined. Intervention group made 27.1% fewer critical errors than control group during on-road assessment	0,89
Marmeleira et al., [48]	*n* = 26 yr = between 55 and 78 years old. c = Portugal	RCT	The main aim of this research was to study the effects of a similar exercise program on the speed of behavior of older adults during on the road driving.	The participants were randomized in 2 groups: -Control group (n:13): Did not receive intervention -Exercise group (n: 13): Was based in an exercises program of 8 weeks with 3 days per week with a session of 60 min. All the participants performed the on-road baseline assessment and after 8 weeks carried out the post-intervention assessment.	(S): Brake Response Time Task: The participants had to brake as quickly as possible whenever the leading car’s rear brake lights were activated. (S): Peripheral Response Time Task: The participants had to react by depressing with their left thumb a microswitch attached to the left side of the steering wheel. (S): Choice Response Time Task: The participants were instructed to follow the leading car and react as quickly as possible to either using the brake or depressing the microswitch on the steering wheel. The leading car’s rear brake lights were activated. (S): Dual-Task Condition: The participants had to brake as fast as possible when the leading car’s rear brake lights were activated and must realize a mental-calculation task.	S (+): The participants under the exercise group showed a significant improvement for the simple, two choice and peripheral reaction time tasks and in the dual task condition. Moreover, a composite score reflecting all reaction time measurements showed a significant improvement.	0,79

**Table 7:** Synthesis of intervention studies involving simulator-based training

Authors	Number (n), Age (yr) Country (c)	R.D.	Objective	Study description	Dependent Variable	Relevant results	QS
Marchal-Crespo et al., [49]	*n* = 32. yr = between 65 and 92 years old. c = USA	RCT	One goal of the present study was, therefore, to determine if the guidance-related learning enhancement persists at a long-term (1 week later) retention test.	Evaluated 4 groups two mainly group divided by age and then every group was randomly assigned in guidance and no guidance: -Guidance group-young (n:15): Drove 15 times with haptic guidance and 5 without. -Guidance group-old (n: 17): Drove 15 times with haptic guidance and 5 without. -No guidance group-young (n: 15): Drove the circuit 20 times without robotic guidance. -No guidance group-old (n:14): Drove the circuit 20 times without robotic guidance The intervention consisted in 3 experimental session on different days. In the first and third session were carried out tests. The second session participants performed the training.	(S): The tracking error: defined as the mean of the absolute value between the center of the simulated wheelchair and the black line, was measured (S): Trajectories followed. (S):Long-term reduction in steering performance. (S): Performance (error reduction).	S (−): Training with guidance significantly improved long-term retention of the task only for younger drivers. Furthermore, improved long-term retention more for initially less skilled drivers and finally improved learning of the steering task in curves, whereas it did not affect learning during straight lines. S (−): Older drivers did not find significant difference in training with guidance or without. S (−): There was an effect of age on driving performance and retention. The older drivers have a worse performance and also learned more slowly and forgot the learned task.	0,75
Rogé et al., [50]	*n* = 31 yr = between 63 and 78 years old. c = France	RCT	Our aim in this study was to test the two following hypotheses: that specific training given during simulated driving would improve elderly drivers’ useful visual field; and that the training given would allow them to detect more easily vulnerable road users than untrained elderly drivers during simulated driving.	The 31 participants were divide in 2 groups: -Experimental (n: 15): training in simulator to increase the useful visual field. -Control (n: 16): driving in the simulator maintaining a constant distance between the vehicle in front. The interventions were based in two visits to the lab separated with 12 days on average. The entire two sessions lasted 5 h and 4 h and 15 min to the experimental and control group respectively.	(S): Useful visual field size: Was estimated during driving. Participants had to detect a change in color (central signal) of a disc which appeared briefly and intermittently on the rear window of the vehicle they were following. 22 central signals appeared during the test. They also had to detect 48 peripheral signals which appeared briefly at 3 eccentricities on the road over 8 different meridians.(S): Visibility distance of vulnerable road users	S (+): There was a significant effect in the useful visual field size, were untrained participants detected a lower number of signals in the central task compared to the trained group. Also, in the peripheral task the experimental group detected a greater number of signals than untrained participants (49,48% vs 27.93%) when this test was administered at the end of the experiment. S (+): The training would allow elderly drivers to improve their ability to detect vulnerable road users while driving. Visibility distance for vulnerable road users was greater in the experimental group than in the control group and the visibility distance was greater in session 2 than in session 1. Also, the type of vulnerable road user also had a significant effect on visibility distance which was greater for pedestrians than for two-wheeled motorized vehicles, were the trained group was better to detect pedestrians in the road environment	0,68

**Table 8:** Synthesis of intervention studies involving mixed training

Authors	Number (n), Age (yr) Country (c)	R.D.	Objective	Study description	Dependent Variable	Relevant results	QS
Porter, [51]	*n* = 54 yr = average age 77.6, 77.1 and 73.6 years old. c = Canada	RCT	The purpose of this study was to examine an alternate form of driver training by utilizing video and global positioning system (GPS) technology, in combination with a classroom-based education program.	The participants were randomized in 3 groups: -Classroom education (n: 18): 55 Alive Mature Driving program, 2 sessions of 4 h. -Video (n: 17): received video and GPS feedback + classroom education. The participant watched the video of their own pre-test drive with the driving instructor and were given very specific instructions on how to improve their own driving -Control (n:19): Not specified The on-road test were performed in the pre and post.	(S): Driving Test (errors): Participants drove a 26 km in a standardized all test were performed with a digital video camera and then were watching to score the driving errors.	S (−): Participants that carried out the 55 Alive Mature Driving program did not significantly change from pre- to post-testing. S (+/−): Participants in the video group and GPS feedback significantly reduced their driving errors after the program. In this group 9 of 17 subjects improved, whereas only 4 of 18 improved in the Education group, and just 1 of 19 improved in the Control group.	0,96
Marottoli et al., [52]	*n* = 126 yr = average age 80.4 and 79.7 years old (ages N/S). c = USA	RCT	This study was designed to determine whether an education program consisting of classroom and on-road training could enhance driving performance.	The participants were randomized in 2 groups: -Classroom + On Road driving training (n: 69): This group received 8 h of classroom and 2 h of on road training. -Control (n: 57): This group receive modules directed at vehicle, home and environmental safety. Both groups performed their training at 8 weeks and finally had driving and knowledge test.	(S): Driving Performance: The road test was based on the Connecticut Department of Motor Vehicles test and assessed a wide range of driving abilities. (SA/K): Knowledge Test: 20 road knowledge questions from the AAA Driver Improvement. Program and eight road sign questions used in our earlier studies. (SA/K): Intervention Participant Perceptions: the participants were asked if they liked the program adherence.	S (+): The program based in a classroom education plus on road driving show some improvements in the driving performance comparing to a control group. After 8 weeks of training the training group was 2.87 points higher than the control group in the road test score. SA/K (+): Moreover, there were difference in knowledge test score after 8 weeks, 3.45 points higher in the intervention than in the control group. Overall, the participants said that they like this type of program and found it beneficial.	0,89
Bédard et al., [53]	n = 75 yr = between 65 and 87 years old. c = Canada	RCT	if the combination of an in-class education program with on-road education would lead to improvements in older drivers’ knowledge of safe driving practices and on-road driving evaluations.	Participants were randomized in 2 groups -Intervention group (n: 38): received the 55-Alive/Mature driving program, as well as 2 sessions of 40-min on road practice. -Control group (n:37): The procedure of this study was the initial on road driving evaluation. 4–8 weeks after completing the training were performed the second on road driving evaluation.	(B): Road evaluation: The on-road evaluation lasted approximately 35 min consisted of varying types of roadways and speeds and left and right turns at controlled and uncontrolled intersections. -Starting/stopping/backing -Signal violation/right of way/inattention -Moving in roadway. -Passing/speed and turning (SA/K): The knowledge was evaluated with a questionnaire that consists of 15 multiple-choice questions	B (+): On the other hand, the on-road evaluation results suggest improvements on some aspects of safe driving such as moving in the roadway. SA/K (+): This study revealed a significant improvement after the education and on road practice, with an increase in the knowledge test from 61% of questions correctly answered at baseline to 81% at follow-up.	0,89
Hay et al., [54]	*n* = 67 yr = average age 75 years old (ages N/S). c = France	NRCT	Compare the effectiveness of two training programs: pure cognitive training and the same cognitive training coupled with three driving simulator training sessions, both programs being addressed to older drivers presenting a cognitive self-assessment bias.	The participants were divided in 2 groups -Cognitive training (CT, n:40): had a duration of 35 h for 12 weeks and was composed of 20 cognitive exercises with 15 difficulty levels each, focused on: attention, memory, visuospatial abilities, executive functions -Cognitive training + driving simulator (CT + DS, n: 27): Was the same training plus 1 h of simulated driving, 3 sessions of 20 min. The evaluations carried out were before and after training trough the cognitive evaluation and on road evaluation.	(The cognitive performance was evaluated with the: (S): Trail Making Test (A and B): assessed processing speed, executive function, and visual scanning ability and involved two parts. (S): Digit Substitution Symbol Test: assessed psychomotor processing speed. (S): The speed of processing and visual attention was evaluated with the Useful of Field of View.(S): The on-road driving evaluation was based on two different, but equivalent road trips combined urban, suburb and rural circuits and a section of ring road/highway. Also, there were two grid, the first, assessed eleven dimensions of driving. The second grid was completed in real time during the trip by the experimenter seated behind the driver.	S (+): Both group of training shows improvement; a diminution in the number of perseverations in the TMT, an increase in the number of correct symbols for the DSST, shorter interval presentation of the target to which they reacted (visual attention), participants anticipated the traffic and the environmental changes better and driving performance. S (+): Participants from the CT group tended to make more planning errors than participants from the CT + DS group, regardless of the time of evaluation S (+/−): The driving simulator experience did not influence the drivers’ behavior on the road. The participants from the CT + DS group did not make significantly fewer driving errors than those from the CT group. Therefore, the addition of driving in a simulator to the cognitive program led to a deterioration in speed adaptation and car control handling performances, whereas the pure CT led to an improvement of these driving performances.	0,82
Casutt et al., [55]	*n* = 91 yr = between 62 and 87 years old. c = Switzerland	RCT	NM	The participants were randomized in 3 groups: -Simulator Training Group (n: 39): A training session took 40 min. The goal of this training approach was to increase the mental work load of correct driving in a realistic multitasking driving setting. -Cognitive Training Group (n: 26): The goal of this training approach was to increase specific driving relevant cognitive functions. Each of the 10 training sessions was composed of 10 min intrinsic alertness training, followed by 10 min of phasic alertness training and 20 min of vigilance training. -Control Group (n: 26): No training. The study design was a pre-post design. During the pre-posttest were conducted the cognitive and on road tests. The experimental groups between the pre-post tests performed the training.	(S): During the on-road test driving assessment the instructors made notes in the evaluation sheet but only evaluating cognitive aspects of driving behavior. Driving performance was measured using driving errors, top speed, mean speed, lane accuracy, lane variability, and reaction time to hazardous events. (S): Cognitive test battery evaluated: Reaction test, Cognitrone test, determination test, peripheral perception test, adaptive tachistoscopic traffic perception test and adaptive matrices test.	S (+): The driving simulator-training group showed an improvement in on-road driving performance compared to the attention-training group and both training groups increased cognitive performance compared to the control group.	0,82
Romoser, [56]	n = 21 yr = “active learning group” age range = 73–82, avg. = 77.4, SD = 3.47 “control group” age range = 72–81; avg. = 76.5, SD = 3.20 c = USA	NRCT	Determine the long-term effects of active training on older drivers’ scanning in intersections.	Participants who participated in the study of Romoser and Fisher (2009) were recruited. -Active learning group (n: 11): Received customized feedback from a replay of his own simulator and field drives evaluations. -Control group (n:10): Received no training All participants performed 6 individual sessions and the training session was the number 4, the other sessions were designated to the pre and post evaluations (simulator and on road).	(B): Videos showing the individual intersection maneuvers of the participants were analyzed to determine if the driver made a correct secondary look at the intersection. The main outcome is percentage of secondary looks, defined as the number of intersections where the driver took a proper secondary look divided by the total number of intersections the driver navigated, was calculated for	B (+): The 2009 study, older drivers in the active learning group took secondary looks in 46.3% of intersections prior to active training in a simulator and in 79.6% of intersections. Two years later, the same active learning group drivers continued to execute secondary looks in intersections 72.7% of the time a result that was still significantly higher than their 2009 pretraining performance. The 6.9% decrease was not statistically significant B (+): Older drivers in the control group who received no training in 2009 took secondary looks in 40.7% of intersections during the first field drive and in 38.5% of intersections 6 to 8 weeks later. Two years later, these same control group drivers took secondary looks in 42.9% of intersections again, no statistically significant change in performance.	0,82
Romoser and Fisher, [57]	n = 54 yr = between 70 and 89 years old (range = 70 to 88; sample mean = 77.54; sample STD = 4.55) c = USA	NRCT	Determine whether older drivers looked less often for potential threats while turning than younger drivers and to compare the effectiveness of active and passive training on older drivers’ performance and evaluation of their driving skills in intersections.	Participants were divided into three age groups (70–74; 75–79; 80–89), then each group were assigned to one of the next 3 groups: -Active learning group (n: 18): Received customized feedback from a replay of his own simulator and field drives evaluations. -Passive learning group (n: 18): Received a traditional lecture-style training session consisting of power points slides, texts, figures and animations. -Control group (n: 18): Received no training All participants performed 6 individual sessions and the training session was the number 4, the other sessions were designated to the pre and post evaluations (simulator and on road).	(B): Videos showing the individual intersection maneuvers of the participants were analyzed to determine if the driver made a correct secondary look at the intersection. The main outcome is percentage of secondary looks, defined as the number of intersections where the driver took a proper secondary look divided by the total number of intersections the driver navigated, was calculated for	B (+): The participants in the active learning group increase their secondary looks more than the double that they took before training and tended to rate this training to be more effective. Between the active and passive groups there was a significant difference, as was that between the active and control groups in their secondary looks. Finally, there were significant differences between active and passive groups and between active and control groups but not between the passive and control groups.	0,81
Lavallière et al., [58]	*n* = 22 yr = between 65 and 85 years old. c = Canada	NRCT	If simulator training, coupled with video-based feedback can modify visual search behaviors of older drivers while changing lanes (Feedback drivers).	The participants were randomized in 2 groups -Feedback group (n:10) -Control group (n:12) Then participated in 5 sessions, where the first and last session included the on road and in simulator assessments. The 3 sessions between the pre-post tests were the same for both groups with the general driver refresher course (based on the 55-alive driver safety) and driving simulator training. The only one difference was that the feedback group received driving specific feedback.	(B): All drivers drove 12 km in the same vehicle on the same open road circuit for both the pre and post-training sessions. For each on-road lane change, 20 s of data were extracted from the records; 15 s prior to the initial displacement of the vehicle towards the target lane and 5 s after this initial displacement. The principal outcomes were: -Frequency of visual inspections during lane changes -Temporal inspection of the blind spot.	B (+): The participants in the feedback group after the training increased the frequency of verification of the blind spot increasing from 32,3% before the intervention to a 64,9% post intervention. Additionally, there was an increase of visual inspections occurring prior to the onset of the lane changes (after the training, 96% of the verifications occurred prior to the onset of the lane changes).	0,75

The dependent variables were categorized according to the methods used to collect them (Table 1): Tests/questionnaires, on-road evaluations, simulator and the combination of all. Also, the dependant variables were grouped to infer on the impact of the given program: Self-awareness and/or knowledge, behaviour, skills and crash rates (Tables 2, 3, 4, 5, 6, 7 and 8).

Self-awareness/Knowledge: Self-awareness of one’s ability to drive and of their capacities and/or limitations to do so safely is mainly evaluated by conducting interviews or via questionnaire. As for knowledge, it is often associated with traffic regulations and laws, as well as the effect of health and other factors on driving.

Behaviour: The drivers’ behaviour is documented as moment of the day, roads used, driven speed, or what has been described by Michon [60] in his model of driving as strategic and tactical levels.

Skills: Described as the operational level of Michon’s model [60], skills are linked with direct control of the vehicle as well as with visual searches surrounding a manoeuvre.

Crash rates (or collisions/accidents): Either collected as self-reported value by participants or by cross-referencing available databases, crash rates (ex. Collisions/accidents per distance driven or per year) are used as a predictor of an intervention’s effectiveness.

## Results

**Fig. 1:**
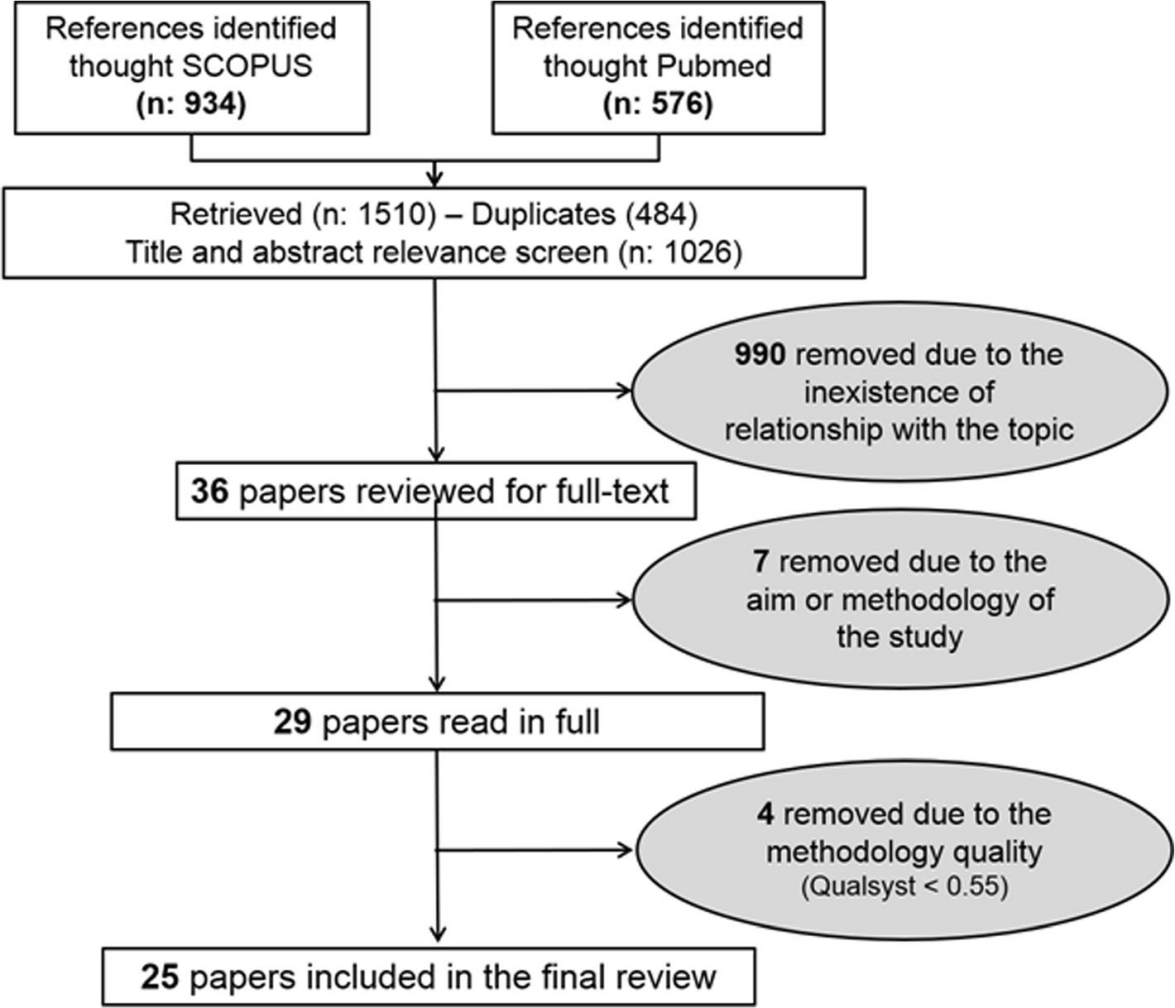
Flow diagram of paper selection process based on PRISMA

Figure 1 shows the results of the search strategy using PRISMA. An initial number of 1510 papers was identified through search of databases (SCOPUS: 934 and Pubmed: 576), from which 484 duplicates were removed. After screening the remaining 1026 title, abstract and keywords of each article, 36 papers were identified as being potentially relevant. Following a complete review of the corresponding full-texts, 29 papers were then selected based on the previously mentioned inclusion criteria. Seven (7) papers were not considered due to different situations [61–67], for example: the objective of the paper by Joanisse et al. [61] was to report the findings from an evaluability assessment of the 55 alive mature driver-refresher course offered by the Canada Safety Council. Another example is the study by Musselwhite [63] where different issues were addressed through an expert group opinion identifying age related physiological and cognitive changes that may be involved in collisions. Finally, after applying the QualSyst [30], 4 papers were removed due to the methodology quality. Thus, 25 papers were included in the final review.

Table 1 shows the summary of studies reviewed. It should be noted that none of the reviewed studies considered route-based or actual driving training as a pure independent variable. Also, it can be seen that 8 of the 25 studies evaluated a combination of more than one type of intervention (Mixed approach), followed by computer-based and education interventions with 7 and 6 studies, respectively. On the other hand, interventions with less studies in this review are those based on physical training and simulator-based training, with 4 and 2 studies each. For more specific information on studies identified in this literature review, detailed descriptions of protocols can be found in Tables 4, 5, 6, 7 and 8.

Tests/questionnaires were used more frequently to evaluate programs, secondly, on-road evaluations and the combination of these two approaches and thirdly, in-simulator alone or combined with on-road evaluation (Table 1).

Table 2 presents the principal dependant variables used to infer on the impact of the given program: Self-Awareness/Knowledge (SAK), Behaviour (B), Skills (S) and Crash Rates (CR). Although 25 studies were reviewed, the number of dependent variables was 28 as three of the studies presented more than 1 dependent variable [35, 52, 53]. The most studied dependent variable was Skills, tested 12 times, followed by studies that considered Behaviour, tested 10 times.

Before presenting the summary of the results (Table 3) and the synthesis of reviewed studies (Tables 4, 5, 6, 7 and 8), it is important to highlight that the effect of the independent variable was classified as (+) when the effect resulted in a significant improvement in the dependent variable, (−) when the effect was significantly negative or no change was observed in the dependent variable and (+/−) when the obtained results were not clear (i.e. non-significant effect or a combination of significantly positive and negative effects on driving).

Overall results show that 60% of reviewed studies presented positive (+) results, 24% presented negative (−), and the remaining 16% of the studies showed unclear results (+/−). For example, from Table 4, the study by Coxon et al. [34] presented unclear overall results (+/−) since self-regulatory driving practices generally showed positive results, but a negative result in the distance driven per week, the restriction of driving space, the use of alternate transportation and community participation 12-months + post-intervention.

Regarding independent variables, the highest overall positive results are for physical training (Table 6) and mixed programs (Table 8) with values of 100 and 88%, respectively. Finally, the lowest overall positive results can be observed when the reviewed studies considered education (43%) and computer-based programs (33%).

In all reviewed studies, primary research approaches were randomized controlled trial (RCT), observed in 17 studies, followed by a non-randomized controlled study (NRCT) and a cohort study used 4 and 3 times respectively. Also, only 1 study presented a pre-test and post-test approach. Finally, regarding sample size, studies evaluated ranged from 11 to 4880 drivers.

### Education-based training programs

Education-based training programs were quite variable in terms of their duration (Table 4), some of them lasting from 1 day up to a full month, the number of classes ranging between 1 and 4. Programs were developed mainly in classroom format. The follow-up evaluation from the intervention was also very variable, going from immediately to 2, 3, 6, and up to 12 months post-intervention. Four (4) of the studies were conducted using a RCT (Level 1) while the remaining 2 used a retro-cohort design (Level 3).

Regarding the dependent variables, 5 of the reviewed studies used questionnaires/tests to evaluate the programs with self-reported driving knowledge, driving behaviours, and driving habits, among others. Drivers reported changing their driving habits following the program and add increased knowledge of road safety facts, but the impact of the intervention faded over time [39]. Only one study used on-road data, measured through collisions and violations of traffic regulations. The remaining study used a mix of questionnaires and monitoring of driving. For the study that evaluated implications in collision [36], drivers who participated in the program add 1.5-time greater odds of being involved in a crash than their matched controls.

### Computer-based training

Table 5 presents reviewed papers regarding computer-based training. Four (4) of the studies were conducted using a RCT (Level 1), 1 used a non-RCT 2-group approach (Level 2), 1 a pre/post-test intervention (Level 3) and 1 a cohort design (Level 3). The most studied dependent variable was assessed through questionnaires/tests (3 of 7 studies). It is also important to acknowledge that 3 of the studies additionally used the on-road assessment or simulator driving for evaluation purposes. Despite differences in the form of their interventions, 4 out of the 7 reviewed studies presented computer-based training based on 10 classes. Follow-up of these studies varied between immediate evaluations, up to 1 to 5 years post-intervention. This later evaluation of the program’s impact was based on crash rates [41]. Two (2) studies assessed behaviours with mixed results on reported outcome, some factors improving such as less driving cessation over 3 years [40] while other specific manoeuvres deteriorated [43]. Speed of processing training showed a positive impact on reducing driving cessation and lowering at fault motor vehicle collisions, as well as improving reaction time [42, 45].

### Physical training

Only 2 studies used physical training to improve driving skills. Both used a RCT (Level 1), 1 using an evaluation scheme similar to an instructor looking at a driver’s overall performance [47] while the second study used different types of evaluations more associated with processing and movement time, such as brake reaction time and peripheral response time tasks [48]. Both programs showed relatively similar active time for the older drivers, Marottoli et al.’s program lasting 21 h [47] and Marmeleira et al.’s program 24 h [48]. The 2 interventions showed a positive impact on driving performance by either maintaining driving capacity after 12 weeks or even improving scores of reaction time measurements. However, it is not possible to identify if there was any issue with adherence to training in these 2 studies.

### Simulator-based training

Of the studies that investigated the effects of simulator-based training on driving performance, only 2 that met the inclusion criteria were reviewed (Table 7). These studies used a randomized controlled trial approach (Level 1), and the dependent variables were gathered using the same simulator. Furthermore, characteristics of the intervention are not very clear, so it is not known how long each class lasted and only one study indicates that the intervention lasted 2 days. Marchal-Crespo et al.’s [49] study showed that, despite being useful for improving driving performance for both younger and older drivers, a guidance system using haptic feedback was not sufficient to transfer learned skills for older drivers and this training was not useful at improving their skills, long-term. Once the guidance system was removed, older drivers returned to their initial driving performance.

Rogé et al.’s [50] study showed a positive effect of their training using a simulator to improve drivers’ useful visual field size. Trained participants showed an improved central and peripheral capacity to detect signals while the control group who drove the same simulator with no specific training did not improve their detection rates.

### Mixed programs

The most studied independent variable in the revised papers was the mixed programs. The most common combination was done by using a classroom setting plus either an on-road intervention with a driving specialist [51–53] or a series of in-simulator interventions with driving-specific feedback combined with active practice in the simulator [57, 58]. Research designs were equally distributed, 4 for each, between Level 1 (randomized controlled trials) and Level 2 (2 or more groups randomly assigned to conditions but not as a RCT). When comparing the types of interventions that received mixed training, a consistent finding is that groups that only received classroom information with no specific feedback and practice of their driving (control groups) did not improve their driving when compared to other combinations of interventions [51, 57, 58]. These results are similar to those observed in the education-based training programs section above.

With the use of a specific data collection system, it is interesting to note that the approaches used by Porter [51], Romoser and Fisher [57] and Lavallière et al. [58] allowed the trainees to receive specific feedback from their own driving performance by using video collected during the initial on-road evaluation.

## Discussion

The purpose of this study was to assess, by a critical review, whether interventions designed for healthy older drivers improve driving on the following components of safe driving: Self-awareness and/or knowledge (SA/K), behaviour (B), skills (S) and/or reduced crash rates (CR). Reviewing the 25 papers selected according to pre-defined criteria, 60% (15 papers) report a positive impact on different levels of driving indicating that interventions are feasible and useful at improving older drivers’ situational awareness/knowledge, behaviours, skills and/or crash rates. However, it is important to mention that some of these results are different from the findings presented by Golisz [59] in their review assessing specific interventions within the scope of occupational therapy practice and including a population with specific health concerns such as stroke survivors [68].

In this section, the primary review findings are discussed separately according to each independent variable (i.e., the effects of education-based training programs, the effects of computer-based training, etc.). The authors realized that the diverse nature of the studies and the variables used in the reviewed studies were quite different, even when testing similar variables, and that different approaches have their own specific strengths and weaknesses.

### Education-based training programs

Education programs present a variety of types of interventions, from the number of classes, number of participants per class, program and class duration. In the current review, the typical class used was based on the 55 alive driving course. This was also observed in mixed training that used part of an education-based approach. On the other hand, when considering the type of class, most interventions were guided by an expert, but there were also programs developed through educational videos, more flexible programs guided by the participants, or simply reading a document. Therefore, it is difficult to propose a “standard” type of intervention or to generate a recommendation on how an intervention based on an education program should be.

Despite the positive findings related to self-regulatory driving practices found in some of the programs [34, 36, 39], the results must be considered with caution since 2 out of 3 studies with positive results related to education programs were informed by self-reports and/or questionnaires [36, 39]. This type of dependent variables could create some problems, for example, in the study developed by Selander et al. [69], all the participants self-reported as capable of driving, however, when evaluated by an objective measurement such as a test on route, 20% of them failed. In another study developed by Freund et al. [70], 38% of the participants were categorized after a simulator test as unsafe drivers, however, all of them self-reported driving performance that was equal or better than other drivers of their age group. Ross et al. [71] found that 85% of older drivers self-reported as being good or excellent drivers regardless of their previous citations or crash rates.

It is also important to highlight that 4 out of 6 of the reviewed studies, including the ones with strong methodology [34, 39], did not produce positive results supporting this type of intervention [37, 38]. Furthermore, the results obtained by Nasvadi and Vavrik [35] indicate that men and women who attended this education course had a 1.5-time greater chance of being involved in a crash than their matched controls. The previous results coincide with Janke’s study [72], which concluded that completing a course of education is not associated with a decrease in crashes after the analysis of 2 cohorts, conversely, those killed and injured in motor vehicle collisions increased. Furthermore, there is a systematic review that indicates that there is no scientific evidence to support the effectiveness of post-licensing education programs in the reduction or prevention of accidents [73]. This could translate into an increased risk of driving since the effects of the education program are not positive and participants feel more confident after participating in a program or maybe that drivers taking these types of classes have concerns about their driving and might already be at risk drivers due to declining skills and/or health conditions.

Overall, despite their widespread use among older drivers and organizations who provide these classes, their proven efficacy to increase driver’s knowledge and self-awareness are not enough to improve one’s ability to drive safely or reduce crashes. Therefore, they should not be used as a single method for an older driver who wants to continue driving. The results obtained from the current review confirm the one from Owsley et al. [74] and McKnight et al. [75]. They showed that educational interventions did not show positive results in improving driving performance and safety, even though drivers increased their knowledge on road safety. Moreover, these results have been confirmed by the mixed interventions identified in the current review showing that with a classroom intervention only, older drivers did not improve their driving performance [51, 52, 57, 58].

### Computer-based training

Despite the huge variability in the methodologies used, only 2 of the 7 studies presented positive overall results on behaviour [40] and skills [45].

Considering the study by Edwards et al. [45], it can be concluded that this type of intervention could positively affect driving mobility, since the participants who completed the processing speed training were 40% less likely to cease driving during the subsequent 3 years, as compared with controls. In addition, another paper showed that the older adults with risk for mobility decline who completed the processing speed training experienced a trajectory of driving mobility similar to the subjects who were not at risk [43]. On the other hand, those who were at risk for mobility decline and did not undergo training experienced greater decrease and difficulty in mobility for the 3 subsequent years. However, those results should be analysed with caution since the dependent variable is based on self-report through the mobility questionnaire. Contrary to the positive findings mentioned above, another reviewed study showed that 5 weeks of training with 2 weekly 60-min sessions guided by a facilitator did not reveal any significant benefit associated with the intervention [46]. This could be due in part to the type of variable used, i.e. a short version of the “Hazard Perception Test”, which corresponds to a more objective tool than the application of a self-reported questionnaire, which has shown a sensitivity of 75% and a specificity of 61% in the ability to predict safe older drivers or dangerous older drivers [76].

Three (3) out of the 7 studies presented unclear results (+/−) [41, 43, 44]. Despite it being important to mention that in the study by Ball et al. [41] 2 out of 3 intervention groups (speed of processing training and reasoning training) reduced at-fault collision involvement over the subsequent 6-year period relative to controls, which would indicate that this type of computer-based program could improves driving performance. This is the only reviewed study on a computer-based program demonstrating an improvement for older drivers.

One of the advantages of computer-based programs are that they can be a great training alternative when considering costs, since today’s access to the Internet has been facilitated, and the widespread use of computers and mobile devices is not a barrier to the implementation of these interventions but rather an opportunity for people to improve their driving skills from their homes [44, 77].

Overall, computer-based interventions are an interesting opportunity for older drivers, since some of them have been shown to reduce the risk of crash involvement over time. However, more research is required to better understand how these interventions improve one’s ability to drive safely, beyond the speed of processing and reduction of reaction time. With computers and smart devices now widely available, training could be done almost anywhere for interested drivers.

### Physical training

For both studies under review in this current analysis, results are positive in terms of impact on driving performance following a physical training intervention aimed at older drivers. The first study showed that physical training allowed older drivers to maintain driving performance over the course of the interventions, while the second program showed improved response time to different secondary tasks while driving. Over 2 different periods of time, 12 vs 8 weeks, they both showed that a regimen equivalent to about 21 to 24 h of exercises could be beneficial to older drivers. Further evaluation should be performed to analyse the best and most efficient modality of interventions with this clientele (ex. Short daily period of exercise or longer period spread across the week).

Moreover, since most interventions aimed at either increasing range of motion and speed of movement, little is known on the impact of cardiovascular training and its transferability to driving capacity. Positive transfer has been shown on cognitive tasks [78] and it would be of interest to evaluate this in a driving context.

### Simulator-based training

Only 2 studies used simulator-based training and presented contradictory results. The negative results reported by Marchal-Crespo et al. [49] showed that older drivers did not benefit from training with haptic guidance, and long-term improvements (1 week) were only observed among younger drivers. On the other hand, Rogé et al. [50] showed that simulator training can improve visual field, allowing older adults to better identify vulnerable users on the road. Dependent variables from the 2 reviewed studies were obtained through the use of simulators, and normally indicate that performance in a driving simulator is strongly related to real driving performance and less to cognitive performance [55]. Therefore, driving tests in simulators could be used to evaluate older adults, as previously suggested by Lee et al. [79–81]. Despite the small number of reviewed studies using simulator-based training as the only intervention, it is interesting to report that in the mixed program, 5 out of 9 studies used simulators with another independent variable [54–58].

Something interesting having repercussions in future research from the study by Rogé et al. [50], is the report of simulator sickness by participants that rendered them unable to continue with the study. Studies using simulators in the mixed interventions below also reported the loss of participants due to simulator sickness. This important issue related to the use of simulator or virtual reality is at the forefront of their widespread use for clinics and programs aimed at older individuals, since they present a higher prevalence of symptoms than their younger counterparts [82]. Fortunately, interventions can be tailored to reduce the importance of such symptoms to allow the driver to accommodate to this new driving environment [83].

### Mixed programs

All the Programs using a mixed approach included specific driving practice either on-road or in-simulator.

Bédard et al. [53] and Marottoli et al. [52] showed that interventions using on-road sessions with an instructor providing specific feedback improved driving scores after the intervention. Unfortunately, the use of general scores to describe a driver’s improvement does not make it possible to extract the specific effect of the intervention or the remaining generalization (ex. moving in the roadway).

Romoser and Fisher [57] compared the effectiveness of active, passive and no training on older drivers’ performance in intersections. Active training in-simulator increased a driver’s probability of looking for a hazard during a turn by nearly 100% in both post-training simulator and on-road driving sessions. Lavallière et al. [58] showed similar results in-simulator with an analysis of visual search strategies during on-road lane changes. Their results revealed that the driving-specific Feedback group increased their blind spot verifications (from 32.3 to 64.9% of the lane changes - an increase of 100%), whereas the control group did not. Porter et al. [51] also used a video intervention but the feedback was not specific to a particular set of driving skills. Porter et al. [51] used a similar paradigm utilizing video and global positioning system (GPS) data, in combination with a classroom-based education program. Their results showed a mitigated impact on driving, since only 9 out of 17 improved their driving by reducing their errors after the program. This difference between Porter et al.’s results and the 2 previous studies might be due to the number of sessions to provide feedback and practice for drivers, since Porter et al. do not report any specific practice of driving weaknesses after receiving feedback.

Only the study conducted by Romoser [56] evaluated the retention of the initial intervention and showed a positive impact 2 years after the program was completed. Only the group who received specific feedback on their driving and appropriate practice in-simulator initially continued to execute secondary looks in intersections prior to turning [57].

For all the studies using customized feedback [51, 57, 58], interventions were successful at modifying drivers’ perception of their driving abilities and positively modifying their subsequent driving skills when returning on-road. The difference between them is that, when particular feedback is aimed specifically at one driving manoeuvre (ex. visual search while turning left [57] or changing lanes [58]), one can expect that these specific manoeuvres should improve after the interventions.

Overall, the mixed approach interventions presented the highest overall score on Qualtsyst and a high frequency of randomized controlled trials (Level 1) (*n* = 4) or multiple groups comparisons (Level 2) (n = 4).

## Limitations of this review

A probable limitation of this review includes the search process itself, which may not have allowed the identification of all studies showing the effects of the different types of programs on driving skills. The use of additional databases such as CINAHL, PsychInfo and ERIC might have led to slightly different results [27].

For the study using a simulator, either as a single intervention or with a mixed approach, simulator sickness still remains an obstacle for the large-scale use of such interventions, in particular if we cannot better understand the underlying mechanisms. The fact remains that some techniques, at best, reduce the incidence and impact of such events on subject participation, and should be used to prevent the prevalence of such impediments on individuals who follow these driving curriculums. For some of the studies, having self-reported collisions as an indicator of driving performance should be used with caution, since self-reports of collision involvement may lack validity [84, 85]. Despite this limitation, this type of reporting remains of interest, as official records might also face an issue of underreporting when addressing older drivers’ involvement for fear of losing their driver’s license [86] when such events are reported to official agencies [87].

Finally, limitation of the systematic review itself is due to lack of consistency when reporting results in training programs aimed at older individuals. The wide variety of research approaches adopted by the reviewed studies also made it difficult to summarize and obtain direct relevant findings, since not all driving parameters were assessed similarly, and the specific driving components that were evaluated were not mentioned. Some of the on-road evaluations used general score checklists similar to the one used by driving specialists [47, 52, 53] while others have assessed specific driving manoeuvres, such as visual inspection during lane change or while turning at intersections [56, 58]. In some studies, it is hard to extract proper information on the used design and method and there is limited reporting on the training program, per se. Moreover, few studies have presented a follow-up evaluation of their program to evaluate the mid or long-term retention of their interventions [56]. Despite identifying 25 interventions aimed at improving older drivers’ performance in hopes of reducing their crash risk, almost all the studies failed to show or did not address collision rate outcomes.

## Conclusion

Overall, the most valuable approaches in terms of specific improvement of driving skills and performance are the ones that have put in place specific training curriculum for every single driver to tackle their specific weakness behind the wheel. This is not surprising, considering the known key-concept defined as transfer-appropriate practice [88]. One must develop his/her own capacity of error-detection in their driving if they want to be able to modify current behaviours and implement appropriate responses.

With the recent development of technology aimed at collecting driving information over longer periods of time (for example, see the SHRP 2 project: www.fhwa.dot.gov/goshrp2), evaluation of one driving’s ability could encompass more and longer driving periods, distance driven and more manoeuvres, allowing for a clearer depiction of what needs to be addressed by a driving instructor or an occupational therapist while developing specific interventions.

Moreover, the availability of computing power and artificial intelligence has brought a new set of tools that allows for automatic detection of driving errors and the possibility to provide automated feedback to the trainees [89]. However, since few of the studies have evaluated the long-term retentions of such interventions [56], future efforts should be made to include this important milestone in their projects. It is of real interest to know when a “refresher” session should be provided to prevent a decrease in performance following an improvement in their abilities [90].

Despite its complex implementation, attempts to combine the most efficient interventions presented in the current review are promising for the development of an efficient way to allow older drivers to maintain and even improve their skills, driving behaviours, and decrease their involvement in motor vehicle collisions.

## Data Availability

The datasets used and/or analysed during the current study are available from the corresponding author upon reasonable request.

## Abbreviations

B: Behaviour
CR: Crash Rates
NRCT: Non-randomized controlled study
PICO: Population, Intervention, Control, Outcomes
RCT: Randomized controlled trial
SAK: Self-Awareness/Knowledge
S: Skills
SLR: Systematic literature review
